# Histone Acetylation in the Epigenetic Regulation of Bone Metabolism and Related Diseases

**DOI:** 10.1155/2021/8043346

**Published:** 2021-07-17

**Authors:** Qinglu Tian, Shiqi Gao, Xuedong Zhou, Liwei Zheng, Yachuan Zhou

**Affiliations:** ^1^State Key Laboratory of Oral Diseases, National Clinical Research Center for Oral Diseases, Department of Pediatric Dentistry, West China Hospital of Stomatology, Sichuan University, Chengdu, Sichuan 610041, China; ^2^State Key Laboratory of Oral Diseases, National Clinical Research Center for Oral Diseases, Department of Cariology and Endodontics, West China Hospital of Stomatology, Sichuan University, Chengdu, Sichuan 610041, China

## Abstract

As the earliest studied epigenetic modification, acetylation has been explored a lot through the years. While bone tissue acts as an indispensable part of body, researches aimed at the relationship between the bone and acetylation became necessary. Some environmental factors like diet may affect the metabolism status that some metabolites especially nicotinamide adenine dinucleotide (NAD) were found able to regulate intracellular histone acetylation in bone metabolism. This review focuses on representing the interaction among acetylation, metabolism, and the bone. The results showed that acetylation connects a lot with bone metabolism, while the explorations about related metabolites like acetyl-CoA or different environmental exposures are still limited. Some acetylation-related therapy methods of bone diseases based on metabolic regulation or epigenetic enzymes were also reviewed.

## 1. Introduction

Epigenetic-metabolic regulation recently became a new topic and definition of a related mechanism between epigenetics and metabolism. Epigenetics is a comprehensive description of mechanisms which control gene expression and cellular activities using multiple modifications without changing the DNA sequence, while many environmental factors like diet, chemical exposure, and temperature may interact with epigenetic regulationvia metabolism.

There are many kinds of macromolecules that take an indispensable part in the regulation of chromatin, such as ATP-dependent remodeling proteins, posttranslational modification (PTM) enzymes, histone chaperones, chromatin recognition proteins, and noncoding RNA [1]. Among all these, PTM gradually come into view in recent years, which mainly refers to proteins that can add or remove modification groups to histones or other proteins. Histone/lysine acetylation is one of the most well-studied posttranslational modification channels in many research fields like cancer, tumorigenesis, skeletal development, cardiovascular disease, and stomatology.

Early studies revealed that hyperacetylation of histones may results in uncompressed chromatin, suggesting a role for histone acetylation in gene activation by increasing the accessibility of DNA to the transcription core. And vice versa, histone deacetylation results in condensed chromatin, leading to gene inactivation [2]. Generally speaking, histone acetylation is usually associated with activation of gene expression, while histone deacetylation is more inclined to downregulate gene expression [3–5].

## 2. Acetylation-Related Enzymes

Enzymes that mediate lysine acetylation and deacetylation were the first identified among PTM enzymes, and there are mainly two big families, histone/lysine acetyltransferase (HAT/KAT) and histone/lysine deacetylase (HDAC/KDAC). In order to express more vividly, in some researches, HDACs are described as “eraser” protein, while HATs are divided into the “writer” protein family based on their effects.

### 2.1. HATs

Many researches implicated the possible therapeutic function of HATs in disease like inflammation, viral infection, solid tumors, and leukemias [1]. So far, the relatively well-studied subfamilies of HATs include histone acetyltransferase 1(HAT1), general control nonrepressible 5(GNC5), P300/CBP-binding protein (PCAF), MYST family, P300, CREB-binding protein (CBP), Rtt109, and TAFII250.

HAT1 was frequently reported to take part in tumorigenesis. In Xia and colleagues' research, the analysis results showed upregulated HAT1 in osteosarcoma (OS), while miR-377 could promote the OS cell apoptosis and even mitigate tumor growth through inhibition of the Wnt signaling pathway via suppressing HAT1 [6]. P300 and CBP are cofactors that they act as partners, and chromatin immunoprecipitation (ChIP) techniques was used to prove that CBP and P300 are localized to promoters of osteoblastic genes during osteoblast differentiation [7]. GATA-binding protein 4 (GATA4) is a protein involved in osteoblast differentiation, which can be acetylated by P300/CBP on lysine residue K313 [8]. P300 can also acetylate PCAF to enhance its HAT activity [9]. And HATs have also been demonstrated to contribute to osteoarthritis and Rubinstein-Taybi syndrome, where mutations in CBP gene may be the reason. [10] PCAF was reported to acetylate proteins like small mother against decapentaplegic (SMAD), NF-*κ*B, and P053, which was also meanwhile regulatable by microRNA. GCN5 is another histone acetyltransferase firstly discovered to link histone acetylation to transcriptional activation, which has a major impact on energy metabolism by sensing acetyl-CoA and acetylating proteins like PGC-1*α* to control genes related to energy metabolism and mitochondrial biogenesis [11]. Moreover, other HATs like the MTST family, Rtt109, and TAFII250 were reported to take part in the regulation of gene activation, signaling, stress response, and metabolism as well [12, 13].

### 2.2. HDACs

The histone deacetylase (HDAC) family has been implicated in removing acetyl groups from lysine residues in histones, as well as other proteins. HDACs are reported to take part in the regulation of gene transcription, protein stability, DNA repair, cytoskeletal dynamics, development and aging, etc. With regard to the bone, there is a wide range of researches reporting that HDACs especially sirtuins play an important part in the development of the bone by influencing bone formation, repair, and regeneration [10, 14], Several osteoblast-related gene including RUNX2, NFATc1, Zfp521, and Pbx1 were reported to be regulated by HDAC-regulatable transcriptional regulators [10]. There are 18 known HDACs within the human genome so far, each of which are distributed into 4 different classes: class I HDACs (HDAC1, HDAC2, HDAC3, and HDAC8), class II HDACs (HDAC4, HDAC5, HDAC6, HDAC7, HDAC9, and HDAC10), class III HDACs, and class IV HDACs (HDAC11) [9, 10]. According to its NAD^+^-dependent enzymatic activity, class III HDACs also have another name—sirtuins [14].

Sirtuin-1 (SIRT1) takes a pivotal role in osteogenesis as a NAD^+^-dependent deacetylase. SIRT1 can influence the acetylation level of Bmi1 gene, and downregulation of SIRT1 was found to be related with osteoporotic phenotype in Bmi-1(-/-) mice, which is further associated with alveolar bone volume [15]. Sun and colleagues drew a similar conclusion by generating a SIRT1 overexpression transgenic mouse model in mesenchymal stem cells (MSCs), indicating that SIRT1 overexpression in MSCs enhanced osteogenesis through epigenetic modification like reducing the acetylation level of forkhead box O3a (FOXO3a), which upregulated expression of FOXO3a and superoxide dismutase 2 (SOD2). Meanwhile, parameters like skeletal size, bone volume, osteoblast number, and alkaline phosphatase-positive areas were all significantly increased [16]. Mills and colleagues found a synergistic interaction of SIRT1 and AMPK on mesenchymal stem cell differentiation [17].

Other sirtuins were proposed to be taking part in bone metabolic regulation as well. In Huh and colleagues' research, during osteoclast differentiation, mitochondrial SIRT3 was proved to inhibit RANKL-mediated osteoclastogenesis in osteoclast precursors derived from SIRT3−/− mice via stabilizing receptor activator of nuclear factor-*κ*B ligand (AMPK) and coordinating PGC-1*β* and estrogen receptor *α* (ER*α*) at the transcriptional level [18]. Fukuda and colleagues' study provided evidence that SIRT7 KO mice developed severe osteopenia due to decreased bone formation along with an increase of osteoclasts. Osterix (OSX) transactivation activity can be upregulated by SIRT7-mediated deacetylation of lysine 368 and influence bone formation via SIRT7 KO mice [19].

## 3. Osteogenesis and Acetylation

As a tissue with an active remodeling process, the bone has been a research hotspot for years. And early researches showed that bone-related parameters like bone volume or strength to a great extent can be influenced by factors like metabolism status or aging. A disordered balance between bone formation and resorption can be manifested as diseases like osteoporosis, increased inclination of bone fracture, bone cancer, and even alveolar bone resorption.

### 3.1. RUNX2 and RANKL: Two Osteogenesis-Related Factors

Osteoblast and osteoclast are two main cell lineages existing in the bone tissue, which are responsible for osteogenesis and osteoclastogenesis, respectively. In recent years, the signaling network of RUNX2 (representative of osteoblast) and RANKL (representative of osteoclast) was actively investigated and so was centrally elaborated as follows. Receptor activator of nuclear factor-*κ*B ligand (RANKL) is secreted by osteoblasts, osteocytes, and stromal cells which bind to its receptor RANK on osteoclasts or its precursors. RANKL is an essential cytokine for the formation, fusion, activation, and survival of osteoclasts [7, 18]. RUNX2, the abbreviation of runt-related transcription factor 2, also known as CBFA1, plays an important role in bone development, and acetylation has been reported to stabilize and promote activities of RUNX2 [20]. In the RUNX2 network, the Wnt/Notch system, SOX9, MSX2, bone morphogenetic protein (BMP), and hedgehog signaling were found to be upstream of RUNX2, while for downstream, osteoblast-related genes such as alkaline phosphatase (ALP), collagenase 1 (MMP1), osteocalcin (OCN), bone sialoprotein (BSP), and osteopontin (OPN) were included [7]. The dysregulation of RUNX2 or RANKL may lead to some bone-related disease and metabolic disorder, too. In osteosarcoma, overexpression of RUNX2 is frequently observed [21].

There were researches revealing the connection between RUNX2 or RANKL expression level and acetylation. Recently, Rojas et al. showed that under myoblastic differentiation, the epigenetically forced expression of RUNX2 and OCN is accompanied by enrichment of the H3K4me3 and H3K27ac marks at the RUNX2 promoter region [22]. According to Ghayor and colleagues, during osteoblast differentiation, acetylation of histone H3 and H4 was significantly enhanced at the promoters of OSX and OCN genes, other osteoblast marker genes, whereas histone deacetylase 1 (HDAC1) recruitment at those promoters was downregulated. Moreover, knockdown of HDAC1 by the short interference RNA (siRNA) stimulated osteoblast differentiation [3]. However, during the induction of osteogenic differentiation, an overall genome-wide loss of acetylation at H3K9/H3K27 and reduction of acetylation occurred at several thousand promoters were detected using ChIP-Seq analysis [23].

### 3.2. Wnt/*β*-Catenin

Wnts are a family of secreted glycoproteins that control cell proliferation, differentiation, and migration. Activation of Wnt may stabilize *β*-catenin by downregulating its phosphorylation level, which further promoted the translocation of Wnts to the nucleus to get to Wnt-targeting genes. And there was recent research showing that Wnt3a stimulates Bmp2 and Alp expression in the osteogenic process [24]. Shares and colleagues reported that histone acetylation of Wnt genes drives osteogenesis, since acetylation increases *β*-catenin activity by masking the ubiquitination sites, which meanwhile reduces the degradation of *β*-catenin and promotes osteogenesis [20]. BMP was proved to play crucial roles in osteoblast differentiation as well, since the downstream target of BMP, Smad1/5, is able to stimulate the mRNA expression of RUNX2 and OSX [24].. SOST (sclerostin), secreted by osteocytes, is known as a Wnt/*β*-catenin antagonist, and prolyl hydroxylase2 (PHD2) is a classic oxygen sensor existing in osteocytes. Stegen and colleagues found enhanced Wnt/*β*-catenin after the deletion of PHD2, which resulted in a high bone mass phenotype at last, and SIRT1-dependent epigenetic regulation of SOST promoter activation may be the underlying mechanism [25].

## 4. Bone-Related Diseases and Potential Therapy Directions

**Figure 1:**
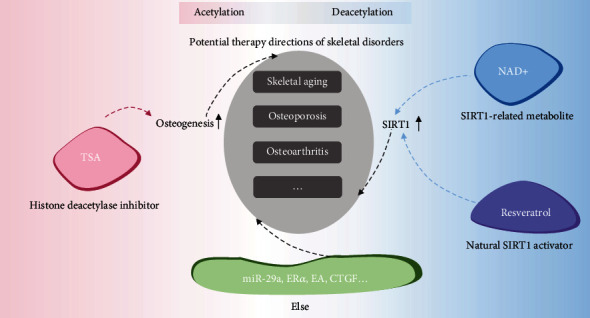
Potential acetylation-based therapy directions of skeletal disorders.

Disorder happening in bone tissue may lead to diseases like bone fracture, osteoporosis, osteoarthritis, and bone cancer, which are common and can be caused by aging, endocrine disorders, or trauma. As the research goes deeper, some potential therapy methods or possibilities based on acetylation were explored and proposed through the years (Figure 1).

### 4.1. NAD^+^: A Key Metabolite in Acetylation-Related Modification

Metabolism is the process that body takes in nutrients from environment and processes them to serve cellular demands, like satisfying the energy demand or regulatory activities, which is indispensable for cell homeostasis. Metabolites are the products of metabolic reactions and can be used as substrates or cofactors for a wide range of epigenome-modifying enzymes, allowing metabolism to directly communicate environmental changes with chromatin state [26]. Under normal conditions, metabolite levels fluctuate around but can be affected by both environmental inputs and intracellular conditions. NAD^+^ is one of the carriers to transport oxygen in the redox reaction of cells. As a signal molecule and a metabolite, NAD^+^ plays a pivotal role in up to 500 enzyme reactions, energy balance, and gene homeostasis maintenance. And NAD^+^ was proved to be the key substrates or cofactors involved in the lysine acetylation and deacetylation reactions [5].

Sirtuins are NAD^+^-dependent HDACs, which means the effectiveness and activity of sirtuins was affected by intracellular NAD^+^ levels. Among all, SIRT1 is the most well-studied sirtuins relating to NAD^+^ metabolism, and quite a lot fields like aging, inflammation, and bone remodeling were included. Nicotinamide phosphoribosyl transferase (NAMPT) is known as the rate-limiting enzyme of NAD^+^ metabolism in the salvage pathway. Imai replenished the concept of “NAD world” in 2016, in which the mechanism between biological robustness breakdown and NAD^+^ metabolism was elaborated and the importance of the inter-tissue communication was emphasized. SIRT1 and NAMPT are two key components in the NAD world, which interact with the hypothalamus, skeletal muscle, and adipose tissue and then promote longevity by regulating metabolism in different body systems [27]. NAMPT overexpression was proved to promote osteogenesis via upregulating the intracellular NAD^+^ level, NAD^+^/NADH ratio, and SIRT1 activity, while adding NAMPT inhibitor FK866 or silencing related genes can lead to opposite results [28–30]. NAD^+^ metabolism could impact osteoclast formation as well. According to Iqbal and Zaidi, CD38-mediated NAD degradation via NADase activity, which discomposes NAD into ADPr, can inhibit osteoclastogenesis by influence RANKL activity. Interestingly, CD38-mediated NAD degradation via ADP-ribolsyl cyclase activity, which discomposes NAD into cADPr, can stimulate osteoclastogenesis [31].

**Figure 2:**
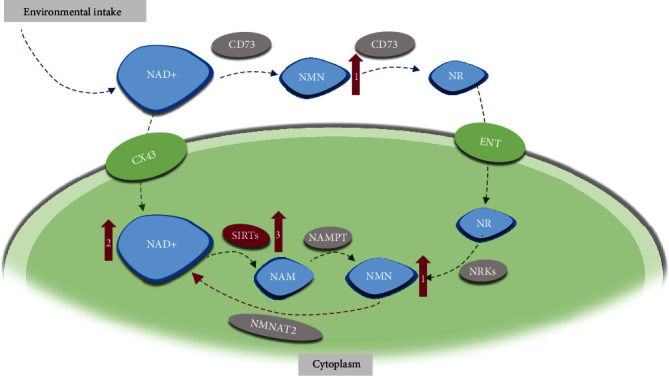
The supplementation of NMN may restore NAD level and SIRT1 activity. The circles filled with gray suggest involved enzymes, while the blue suggest related intermediates. The red arrows with a number on it illustrate the influence of NMN supplementation in order. NMN: nicotinamide mononucleotide; NR: nicotinamide riboside; ENT: equilibrative nucleoside transporters; CX43: connexin 43; NAMPT: nicotinamide phosphoribosyl transferase; NRK: nicotinamide riboside kinase 1; NMAT2: nicotinamide mononucleotide adenylyltransferase; NAM: nicotinamide.

Supplementation of metabolites/intermediates in the metabolic network like NAD^+^ became a new direction for bone disease treatment recently. The detailed description of NAD metabolic networks can be found in Imai and Guarente' s review [32], and a simplified schematic diagram is displayed (Figure 2). Nicotinamide mononucleotide (NMN) is one of the intermediates during NAD^+^ biosynthesis, which can be obtained in various natural foods like broccoli and cucumber. After long-term administration of NMN, a significant increase of bone density was observed [17]. And Song and colleagues proposed to supplement NMN to restore NAD level and SIRT1 activity as a potential therapy for skeletal aging and osteoporosis [33].

### 4.2. Resveratrol: A Natural Activator of SIRT1

The typical deacetylase activators introduced here is resveratrol, a stilbenoid and a natural activator of SIRT1, which can be obtained from common plant foods like red grapes and blueberries.

Resveratrol was proposed as a novel and potential treatment strategy for osteoporosis and osteoarthritis in researches. While applying resveratrol, the results revealed the promotion role of SIRT1 in MSC proliferation and osteogenic differentiation, and adipogenesis was inhibited [15, 34]. The underlying mechanism may be the upregulation of RUNX2 and OCN accompanying suppression of adipolineage genes PPARg2 and LEPTIN. Importantly, an obvious increase of SIRT1-FOXO3a complex was observed, which may enhance RNUX2 gene transcription activity [34]. And according to Deng and colleagues, activating SIRT1 via resveratrol with improved bioavailability may be an appropriate therapy approach for osteoarthritis (OA). [35] Asis and colleagues put forward that more attention should be devoted to resveratrol analogs in future studies due to its effectiveness, relatively low cost, and low risk [36].

In recent years, resveratrol began to make its mark in the field of stomatology. Resveratrol was proved to inhibit inflammatory responses like enhancing heme oxygenase-1 (HO-1) expression via nuclear factor E2-related factor 2 (NRF2) signaling, which mitigated ligature/lipopolysaccharide- (LPS-) mediated alveolar bone loss in rats in an experimental periodontitis (EP) model as a result [37]. Similarly, Ribeiro and colleagues confirmed that resveratrol inhibits EP and cigarette smoke inhalation- (CSI-) induced loss of supporting alveolar bone and has a beneficial effect on osteoimmune-inflammatory markers [38]. And the potentials of resveratrol like improving bone healing processes in dental implants cases or jaw osteonecrosis caused by bisphosphonates were mentioned in Murgia and colleagues' review [39].

### 4.3. TSA: A Histone Deacetylase Inhibitor

Histone deacetylase inhibitors (HDIs) were explored a lot as a potential therapy choice for bone-related disease. Among all, trichostatin A (TSA), a histone deacetylase inhibitor, is proved to be effective in many epigenetic-metabolic regulation activities of the bone in recent years. It can enhance human periodontal ligament cells (hPDLCs) osteogenesis, and meanwhile, the hyperacetylation of RUNX2 is also observed while applying TSA [40]. According to Ghayor and Weber, TSA can lead to enhanced acetylation of histone H3 and H4, which is related to the increase of RANKL promoter activity in murine bone marrow stromal cells [3]. Keil and colleagues' research revealed that while promoting histone acetylation by applying TSA, increased Bmp2 and 4 mRNA abundance and an extensive branching phenotype in mouse urogenital sinus (UGS) explant cultures was observed, which may give us a hint of a potential role of acetylation in mesenchyme-derived tissue branching morphogenesis [4]. In 2017, the researchers explored whether HDAC inhibition prevents Klotho loss and mitigates the chronic kidney disease- (CKD-) associated bone disorder in a mouse model of CKD-MBD. Intriguingly, TSA prevented Klotho loss by increasing the promoter-associated histone acetylation, therefore increasing Klotho transcription, suggesting the therapeutic potentials of endogenous Klotho restoration by HDAC inhibition in treating CKD and other complications [41]. It was also shown in a recent study that the treatment of nonosteogenic cells with TSA allows Wnt3a to promote osteogenesis in these cells, suggesting that direct conversion of nonosteogenic cells into osteoblastic cell types without inducing pluripotency might be controlled by histone modifiers [3]. And according to Cho and colleagues, direct conversion of nonosteogenic cells into osteoblastic cell types without inducing pluripotency may largely improve prospects for novel epigenetic therapies to treat skeletal disorders [24].

### 4.4. Other Potential Therapy Directions

Osteoporosis is a common bone-related disease characterized by decreased bone mass and adipocyte accumulation within the bone marrow that inhibits osteoblast maturation, leading to a high risk of fractures [3, 36, 42]. Estrogen receptor *α* (ER*α*) is an important modulator of bone homeostasis, and it is well established that estrogen deficiency induces osteoporosis. Moon and colleagues' research revealed that SIRT6-mediated deacetylation can increase ER*α* level in preosteoclasts to prevent bone loss by inhibiting osteoclast-mediated bone resorption [43]. To speak at RNA level, miR-29a was found to suppress PCAF-mediated acetylation and further reduce osteoclast differentiation by regulating RANKL and CXCL12 against osteoporosis [44]. It was also reported that miR-29a can increase acetylated RUNX2 and *β*-catenin abundances and reduce RANKL, leptin, and glucocorticoid receptor expressions in bone tissue microenvironments via inhibition of the histone deacetylase 4 (HDAC4) expression, which may mitigate glucocorticoid-mediated skeletal disorders [45]. Other ways like electroacupuncture (EA) was proved to downregulate the expression of HDAC2 and promoted the acetylation of histone H3 as well, which lead to improvement of the bone mineral density and trabecular morphology in ovariectomy-induced osteoporosis [46]. According to Kim and his colleagues, connective tissue growth factor (CTGF) may regulate P300 acetyl transferase meditated acetylation to increase RUNX2 protein stability and meanwhile can promote RUNX2 recruitment to the RANKL promoter, resulting in increased RANKL production from the tumor cells [47].

## 5. Conclusion

Epigenetics becomes a hot research field as the mysterious mask of gene landscape being gradually uncovered. Despite the digging direction towards “deeper structure” of cells or chromatin, the influences of environmental factors especially some metabolites were reconnected to intracellular molecules. Posttranslational modifications act as the “bridge” connecting the intracellular world and outside environment. As a pivotal modification way, acetylation was actively studied, and the relationship between the bone and acetylation has been widely committed. In the field of bone, researches about HDACs especially sirtuins are relatively mature and ample, while the discussions of HATs seem comparatively insufficient. Among all, SIRT1 is the most thoroughly studied. The abovementioned factors consist of the epigenetic-metabolic regulation network of the bone.

The bone tissue can be easily affected by metabolism status, and diseases like osteoporosis are closely linked to metabolic disorder. For disease treatment, there is no absolute conclusion that we should promote or weaken acetylation level, on account of that it depends on the type or cause of the disease itself and on the target of therapy methods (Figure 1). For instance, in the case of osteoporosis, SIRT6-mediated deacetylation and downregulation of HDAC2 were both proved to be helpful to mitigate osteoporosis, while the former led to increased ER*α* level and the latter led to promoted acetylation of histone H3 [43, 46]. Substance like NAD^+^ or its intermediates, resveratrol, and TSA were studied as a potential therapy choice for bone-related diseases based on their interaction with HDACs. Some of them can be easily obtained from common diet, although there is no applicable medicine in the market so far. And we can notice a relatively lack of researches between possible therapy and HATs, which left future studies a space and direction.

**Figure 3:**
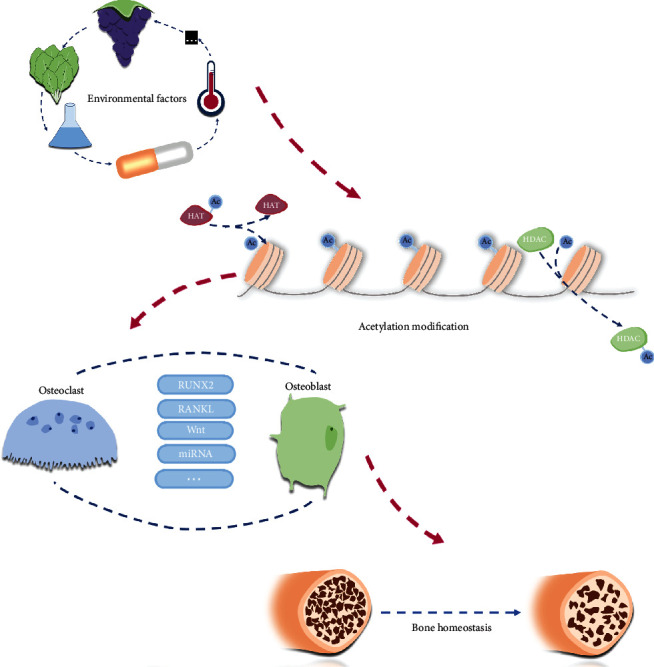
The cascade relationship of environment, acetylation, and bone homeostasis.

To sum up, there is indeed an epigenetic-metabolic regulation pattern existing between the bone and acetylation. Future studies are still needed to complete the missing puzzles like a more specific mechanism and a wider range of metabolites. And this kind of pattern also sheds a light on that crosstalk of macroscopic and microcosmic researches might be a trend (Figure 3).

## Abbreviations

NAD: Nicotinamide adenine dinucleotide
PTM: Posttranslational modification
HAT: Histone acetyltransferase
KAT: Lysine acetyltransferase
HDAC: Histone deacetylase
KDAC: Lysine deacetylase
HAT1: Histone acetyltransferase 1
GCN5: General control nonrepressible 5
PCAF: P300/CBP-binding protein
OS: Osteosarcoma
CBP: CREB-binding protein
SMAD: Small mother against decapentaplegic
ChIP: Chromatin immunoprecipitations
GATA4: GATA-binding protein 4
SIRT: Sirtuin
MSC: Mesenchymal stem cell
FOXO3a: Forkhead box O3a
SOD2: Superoxide dismutase 2
RANKL: Receptor activator of nuclear factor-*κ*B ligand
AMPK: AMP-activated protein kinase
ER*α*: Estrogen receptor *α*
OSX: Osterix
RUNX2: Runt-related transcription factor 2
SOX9: Sex determining region Y- (SRY-) related high-mobility group (HMG) box 9
MSX2: Muscle segment homeobox 2
BMP: Bone morphogenetic protein
ALP: Alkaline phosphatase
MMP1: Collagenase 1
OCN: Osteocalcin
BSP: Bone sialoprotein
OPN: Osteopontin
siRNA: Short interference RNA
SOST: Sclerostin
PHD2: Prolyl hydroxylase2
NMN: Nicotinamide mononucleotide
NR: Nicotinamide riboside
ENT: Equilibrative nucleoside transporters
CX43: Aberrant connexin 43
NAMPT: Nicotinamide phosphoribosyl transferase
NRK: Nicotinamide riboside kinase 1
NMAT2: Nicotinamide mononucleotide adenylyltransferase
NAM: Nicotinamide
OA: Osteoarthritis
HO-1: Heme oxygenase-1
NRF2: Nuclear factor E2-related factor 2
LPS: Ligature/lipopolysaccharide
EP: Experimental periodontitis
CSI: Cigarette smoke inhalation
HDI: Histone deacetylase inhibitor
TSA: Trichostatin A
hPDLC: Human periodontal ligament cell
UGS: Urogenital sinus
CKD: Chronic kidney disease
EA: Electroacupuncture
CTGF: Connective tissue growth factor.
